# Follicular unit extraction combined with fractional carbon dioxide laser therapy for post‐traumatic eyebrow scar with defects: A prospective and comparative study

**DOI:** 10.1111/jocd.16518

**Published:** 2024-08-11

**Authors:** Ping Xue, Qing Yang, Lei Guo, Yue Yin, Wenjie Dou, Tong Li, Xianhui Zeng, Baoqiang Song, Xing Fan

**Affiliations:** ^1^ Department of Plastic and Reconstructive Surgery, Xijing Hospital Fourth Military Medical University Xi'an China

**Keywords:** eyebrow defect, follicular unit extraction, fractional carbon dioxide laser

## Abstract

**Background:**

Eyebrows play a crucial role in the human body. While Follicular Unit Extraction (FUE) is a widely utilized and highly effective treatment for typical eyebrow deficiencies, it may not yield satisfactory outcomes for patients with post‐traumatic eyebrow scars and defects.

**Objective:**

A prospective comparative clinical study was conducted to explore the treatment outcomes of post‐traumatic eyebrow scars accompanied by defects using a combination of ablative fractional CO_2_ laser therapy with FUE.

**Method:**

Between January 2019 and January 2023, we enrolled 30 patients with post‐traumatic eyebrow scars and accompanying eyebrow defects, randomly assigning them to experimental and control groups. Patients in the control group received FUE treatment exclusively, whereas patients in the experimental group underwent CO_2_ fractional laser therapy on the eyebrow scars prior to FUE treatment. Alongside the patients' baseline data and the quantity of transplanted follicular units during surgery, we will compare the follicular survival rates between the two groups at 6 and 12 months post‐treatment.

**Results:**

Prior to FUE, there were no notable variances in baseline data between the two‐patient groups. At 6 and 12 months postoperatively, the follicular survival rate in the experimental group was significantly higher compared to the control group. Additionally, patients in the control group were more prone to experiencing postoperative asymmetry between their eyebrows and developing curly hair.

**Conclusion:**

For patients with post‐traumatic eyebrow scars accompanied by eyebrow defects, we applied ablative fractional CO_2_ laser therapy in combination with FUE treatment. This approach not only resulted in a higher follicular survival rate postoperatively but also led to the achievement of a well‐defined eyebrow shape.

## 1 INTRODUCTION

Hair holds significant importance for humans, with eyebrows arguably holding more significance than other body hair.^1^ While baldness may be perceived as a symbol of normalcy, even maturity, and composure, the absence of eyebrows can lead to feelings of awkwardness and unnatural appearance. Compared to other primates, humans have relatively sparse hair, yet eyebrows begin to develop in the fetus at 9 weeks.^2^ In addition to their physiological functions such as blocking sweat from entering the eyes and shielding from intense sunlight, eyebrows are an indispensable component of facial aesthetics.^3^ If eyes are considered the windows to the soul, conveying nonverbal information like books, then eyebrows can be likened to the cover of those books. Research further indicates the significant role eyebrows play in facial recognition. Even when the eyes are removed from celebrity photos, 56% of participants can accurately identify the individuals, but when the eyebrows are removed, this percentage decreases to 46%.^4^

There are various causes of eyebrow defects, including genetic disorders, endocrine diseases, and trauma.^5^, ^6^ In clinical practice, we predominantly encounter eyebrow defects resulting from trauma and burns. Traditional treatment methods for eyebrow defects have mainly involved pedicle flap grafting, which requires high technical proficiency and involves lengthy treatment periods.^7^ However, with the continuous advancement of hair transplantation techniques, hair transplants have become the primary approach for treating eyebrow defects. Among these techniques, FUE has become one of the most common methods due to its advantages of minimal trauma, rapid healing, absence of linear scarring, and no need for sutures compared to other follicular transplantation techniques.^8^ Nonetheless, the postoperative follicular survival rate in patients with post‐traumatic eyebrow scars accompanied by eyebrow defects remains unsatisfactory. This may be related to the impact of scars on the surgery, and lasers can improve scar tissue hardness and reorganize collagen fibers. Therefore, for patients with post‐traumatic eyebrow scars accompanied by eyebrow defects, we are undertaking a study exploring the effects of combining laser therapy with hair transplantation.

## 2 PATIENTS AND METHODS

This study adheres to the Helsinki Declaration and ethics committee approval was obtained, with all patients having signed informed consent. From January 2019 to January 2023, a total of 30 patients were enrolled in the study. The inclusion criteria for patients were: (1) presence of eyebrow defects with scars due to trauma; (2) aged between 18 and 60 years; (3) scar formation from trauma exceeding 1 year; and (4) provision of informed consent. The exclusion criteria for patients were: (1) presence of chronic diseases that could affect treatment; (2) previous laser or related treatments for eyebrow scars; and (3) patient withdrawal during the study. Using a random number table method, the 30 enrolled patients were randomly divided into two groups, with 15 patients in the experimental group and 15 patients in the control group. Patients in the experimental group underwent carbon dioxide fractional laser therapy on the eyebrow area before receiving eyebrow follicle transplantation, while patients in the control group underwent eyebrow follicle transplantation directly after the maturation of the eyebrow scars.

### 2.1 Laser treatment

For patients in the experimental group, prior to the follicle transplantation procedure, we utilized the fractional ablative CO_2_ laser (CO2RE, Syneron Candela) with a wavelength of 10 600 nm to promote dermal regeneration, improve the texture, smoothness, and elasticity of scar tissue. The treatment was conducted three to six times, with a 3‐month interval between each session, using the fusion mode with energy ranging from 55 to 60 J/cm^2^.

### 2.2 Eyebrow transplantation

#### 2.2.1 Preoperative preparation

One day before the surgery, the patient's eyebrow shapes were designed and marked, estimating the required number of follicles for transplantation, typically ranging from 100 to 400 single hair follicles for unilateral eyebrow defects. On the morning of the surgery day, patients were instructed to wash their hair, and 1 h before the procedure, they were administered 0.2 g of celecoxib. The donor sites for the surgery generally included the occipital scalp, posterior neck, and peri‐auricular hair.

#### 2.2.2 Surgical technique

**FIGURE 1:**
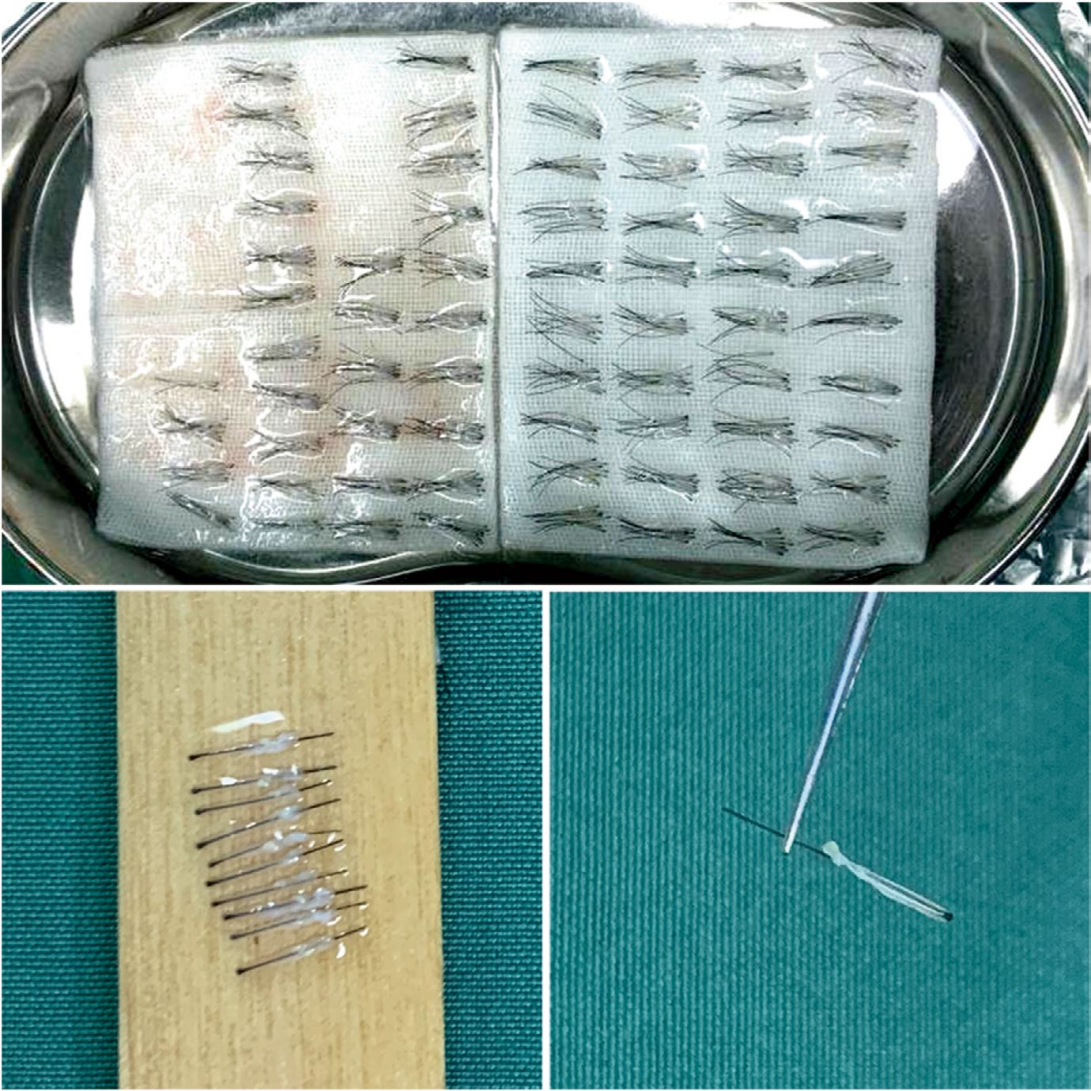
The image shows well‐separated hair follicles extracted during a FUE transplant procedure. Upper: the hair follicle units extracted by low‐temperature hydrolysis; left lower: a bundle of follicles containing 10 individual follicular units; right lower: one individual follicular unit.

When extracting follicles, patients are positioned in a prone or lateral decubitus position, and the donor area is disinfected with iodine. A swelling solution containing 5 g/L lidocaine, 1.875 g/L bupivacaine, 10 mL of saline, and epinephrine hydrochloride solution is prepared in a 1:100 000 ratio to form a 20 mL solution for local anesthesia of the donor area. Follicles are extracted using a 0.8 mm diameter punch, ensuring at least one follicle unit spacing between two punch holes. Extracted follicles are placed on gauze soaked in saline solution on a curved dish above ice to maintain a low temperature (Figure 1).

Subsequently, follicles are dissected, with all follicles separated into single‐hair units. Excess skin and fat tissue surrounding the follicles are removed. During dissection, ice saline solution is regularly sprayed on the dissection board to ensure follicle hydration at a low temperature. Dissected follicles are classified by thickness and arranged in groups of ten on gauze for convenient counting.

When performing follicle transplantation in the eyebrow area, patients are placed in a supine position, and local anesthesia is administered using a mixture of 10 g/L lidocaine and 3.75 g/L bupivacaine in a 10 mL solution with 1/100 000 epinephrine. Concurrently with the anesthesia injection, methylene blue is used to mark the outline of the eyebrow.

**FIGURE 2:**
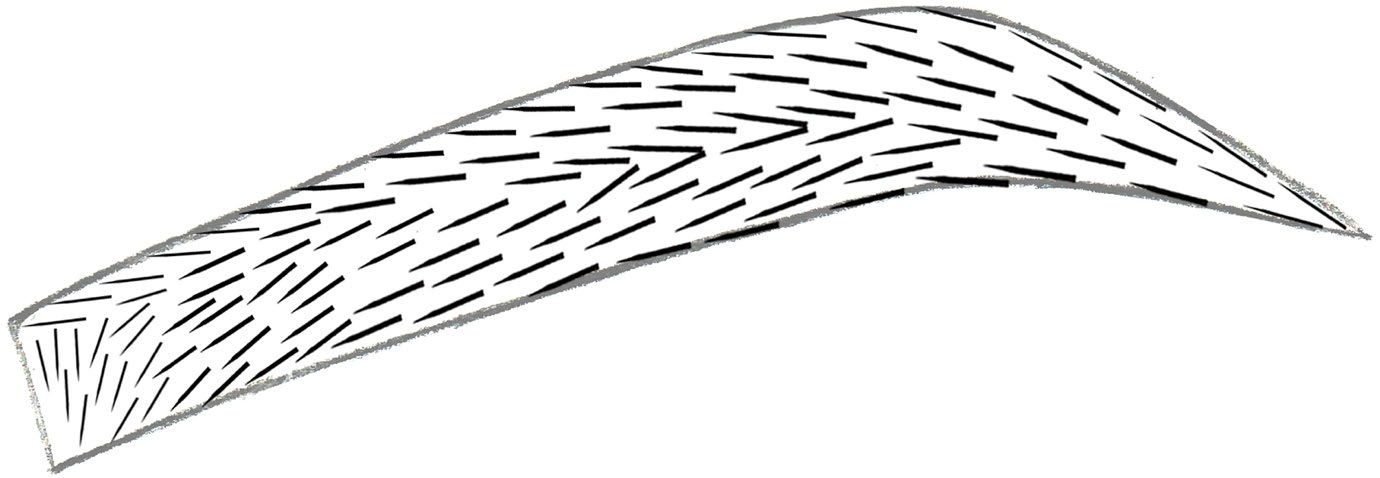
Diagram illustrating the direction of puncture with the punch needle in the donor area during eyebrow transplantation.

Due to the varied growth directions and angles of hair in different parts of the eyebrows, the direction of the incisions made during transplantation also varies. For the frontal region of the eyebrow, the incision direction transitions gradually from vertical or outward slanting downward to inward slanting downward. The upper region of the eyebrow body has an incision direction slanting upward inward, while the lower region transitions from inward slanting downward to horizontal. In the tail region, the upper portion has an incision direction slanting upward inward, while the lower portion is horizontal (Figure 2). These are general guidelines based on experience with most patients. However, for each individual case, adjustments must be made based on the direction of residual eyebrow hair growth, as well as the patient's gender, age, and preferences regarding eyebrow shape.

We typically use a 19–21 G injection needle for making incisions, with the tip bent at a 120° angle to facilitate the procedure. The angle of insertion is maintained at 15°–30° with respect to the skin, and adjusted according to the actual direction of hair growth. The depth of the incisions is determined by the length of each follicle, generally ranging from 4.2 to 4.5 mm. Each needle is replaced after making 10–20 incisions to ensure straight perforations. After follicle transplantation, the recipient area is rinsed with saline solution, followed by applying saline‐soaked gauze for moist dressing and then secured with dry gauze bandaging.

#### 2.2.3 Postoperative care

The patients are required to visit the clinic on the first‐day post‐operation to rinse the surgical area with saline solution. Subsequently, within the following 3 weeks, patients are instructed to cleanse the area daily, spraying it with saline mist every 2 h to keep it moist. During the first 3 days post‐operation, the peak swelling period, oral corticosteroids (dexamethasone, 3 mg per dose, once daily) are given. Additionally, recombinant FGF gel (produced by Zhuhai Yisheng Biopharma Co., Ltd.) is applied topically twice a day for 3 weeks.

Starting from 1 week postoperatively, the transplant area is treated with 5% minoxidil solution (manufactured by Shanxi Zhendong Ante Biopharmaceutical Co., Ltd.), applying 0.3 mL per use once daily. The application continues until the transplanted hair has grown substantially, typically within 3–6 months, after which the medication is gradually discontinued.

### 2.3 Assessment item

The items we need to collect include (1) patient baseline data such as age, gender, patient and observer scar assessment scale (POSAS), which includes physician assessment of scar vascularization, pigmentation, pliability, thickness, and relief, and patient self‐assessment of scar pain or itchiness, scar integrity, irregularity, as well as differences in thickness and color. Each item is scored from 1 to 10, with 1 representing normal skin and 10 representing the most severe condition. The total score is calculated for both physician assessment and patient self‐assessment and laser treatment time; (2) number of follicles needed for transplantation during surgery; (3) survival rate of transplanted follicles at 6 and 12 months postoperatively; and (4) postoperative complications.

### 2.4 Statistical analysis

The data were analyzed using SPSS 26.0 (IBM Statistics for Windows, IBM Corp. Armonk, NY, USA). Means or medians were used to represent the data for the two patient groups, and differences were analyzed using the Kruskal–Wallis *H* test. A significance level of *p* < 0.05 was considered indicative of a statistically significant difference.

## 3 RESULTS

The average age of patients in the experimental group was 37.33 ± 7.89 years, with 12 males and three females, while in the control group, the patients had an average age of 35.80 ± 9.60 years, with 14 males and one female. The scar condition of the experimental group patients before and after laser treatment was evaluated using POSAS. The observer rating for experimental group patients before laser treatment was 17.0, and the patient self‐rating was 19.0; after laser treatment, the observer rating for experimental group patients was 5.0, and the patient self‐rating was 11.0. The observer rating for control group patients was 15.5, and the patient self‐rating was 18.0. There were no significant differences in baseline data between the two groups before treatment. The average number of laser treatment sessions for patients in the experimental group was 4 (Table 1).

**TABLE 1:** Patient baseline data.

	Experimental group	Control group
Age, years	37.33 ± 7.89	35.80 ± 9.60
Sex	12 M, 3 F	14 M, 1 F
Pre‐laser treatment POSAS		
Observer assessment	17.0 (15.0, 25.0)	15.5 (11.0, 23.0)
Patient assessment	19.0 (15.75, 22.5)	18.0 (15.0, 25.0)
Post‐laser treatment POSAS		
Observer assessment	5.0 (3.0, 13.0)	\
Patient assessment	11.0 (8.0, 24.0)	\
Laser treatment sessions	4 (3, 5)	\

*Note*: Age is expressed as mean ± standard deviation, while scar score and number of treatment sessions are represented as median and interquartile range.

Abbreviations: F, female; M, male; POSAS, patient and observer scar assessment scale.

In terms of the number of follicles transplanted during surgery, the average number of transplanted follicles was 454.67 in the experimental group and 501.33 in the control group, with no significant difference between the two groups. However, the follicle survival rates at 6 and 12 months post‐surgery for the experimental group patients were 86.13 ± 5.01% and 81.87 ± 5.26%, respectively, while for the control group patients, the follicle survival rates at 6 and 12 months post‐surgery were 78.07 ± 7.82% and 74.00 ± 7.46%, respectively. There were significant differences between the two groups, with *p*‐values of 0.003 and 0.002, respectively (Table 2). We also assessed the postoperative complications in both groups of patients, and it can be observed that the occurrence of hair curliness and asymmetry on both sides was significantly higher in the control group compared to the experimental group (Table 3).

**TABLE 2:** Comparison of follicle transplant quantity and follicle survival rate.

	Experimental group	Control group	*p*‐value
Follicle transplant quantity	454.67	501.33	0.455
Follicle survival rate %			
6 months post‐op	86.13 ± 5.01	78.07 ± 7.82	0.003*
12 months post‐op	81.87 ± 5.26	74.00 ± 7.46	0.002*

**TABLE 3:** post‐operative complications.

	Experimental group	Control group
Bleeding	1	1
Infection	1	0
Neuralgia	1	1
Hair curling	2	11
Asymmetry	1	5

### 3.1 Cases report

#### 3.1.1 Case 1

**FIGURE 3:**
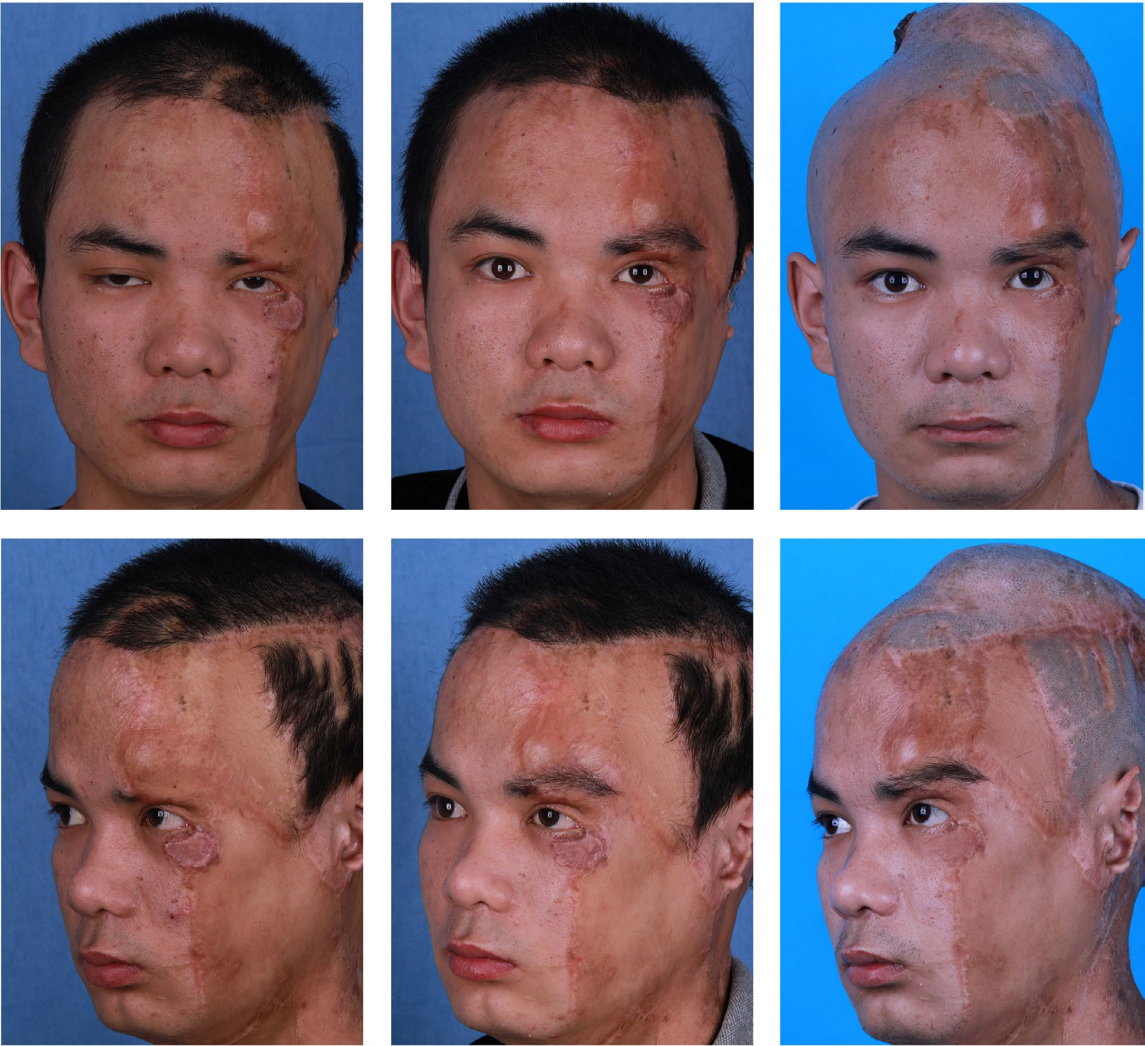
Treatment of eyebrow deficiency in a patient with facial burns using a combination of carbon dioxide fractional laser and hair follicle unit extraction and transplantation. Left upper and lower: before treatment; right upper and lower: 6 months post‐treatment; right upper and lower: 1 year post‐treatment.

A 20‐year‐old male patient suffers from eyebrow scarring and alopecia due to burns, significantly impacting his daily life. Therefore, he sought eyebrow transplantation to improve his facial appearance. The observer rating and patient rating on POSAS before laser treatment were 19 and 21, respectively. He underwent a total of three laser treatment sessions, resulting in significant improvement in the scar condition after treatment. The observer rating and patient self‐rating on POSAS after improvement were 8 and 14, respectively. During the surgery, 245 follicles were transplanted, with follicle survival rates at 6‐ and 12‐month post‐surgery of 83% and 78%, respectively. The surgical outcome was satisfactory, with eyebrow follicle direction and density meeting expectations (Figure 3).

#### 3.1.2 Case 2

**FIGURE 4:**
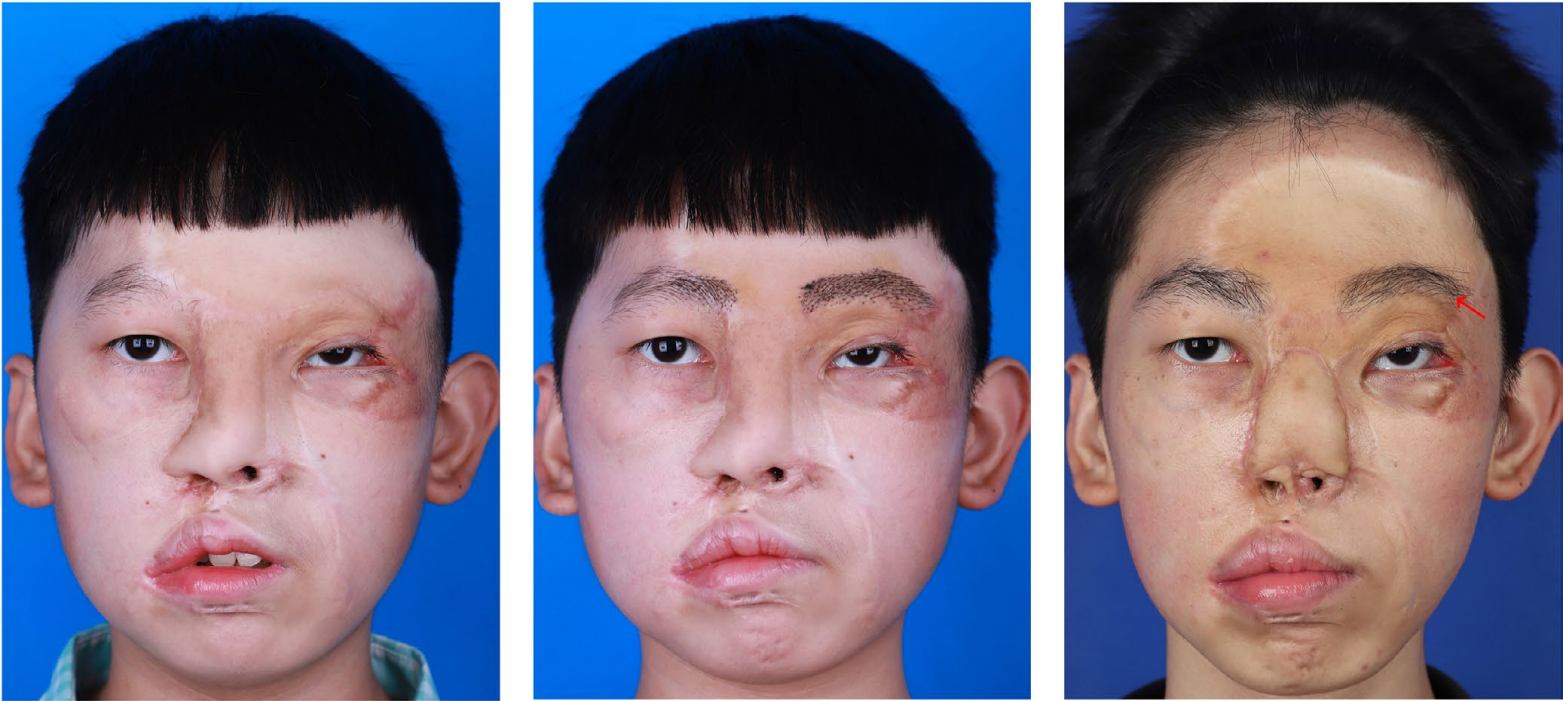
Treatment of eyebrow deficiency in a patient using hair follicle unit extraction and transplantation. Left: before treatment; middle: 1 week post‐treatment; right: 2 years post‐treatment. The transplanted area of the eyebrows shows significant sparseness, with some eyebrows curling (as indicated by the red arrows in the image).

A 13‐year‐old male patient experienced eyebrow loss 2 years ago due to a severe traffic accident. The patient presents with left eyebrow and partial right eyebrow loss following scar repair with facial flap transfer surgery. He observer rating and patient rating on POSAS were 17 and 22, respectively. During the surgery, 485 follicles were transplanted, with follicle survival rates at 6‐ and 12‐months post‐surgery of 79% and 73%, respectively. The surgical outcome was acceptable; however, there was a noticeable asymmetry between the eyebrows, and the hair appeared curly, deviating from the appearance of normal eyebrows. This could be related to the firm texture of the scar tissue (Figure 4).

## 4 DISCUSSION

The causes of sparse or absent eyebrows are diverse, but the most common encountered in clinical practice is eyebrow deficiency following trauma. Deep‐seated traumas inevitably result in scar formation upon healing. Scar tissue, compared to normal healthy skin, is akin to barren land, characterized by stiff tissue, poor blood supply, and susceptibility to infection.^9^, ^10^ Therefore, the survival rate of transplanted follicular units under such conditions is not ideal, similar to how seeds require fertile soil to root, sprout, and grow robustly. The essential foundation for successfully generating healthy hair shafts on scars through hair transplantation is that the scar tissue being transplanted must be in a mature stage, possess a certain level of elasticity and flexibility, and have sufficient vasculature to ensure transplant survival. One of the functions of laser scar treatment is to induce inflammation by generating heat, thereby increasing vascular permeability, stimulating matrix metalloproteinase production, and promoting the decomposition and reorganization of collagen fiber bundles, ultimately leading to improvements in scar thickness, flexibility, and appearance.^11^ Therefore, we aim to perform laser therapy on eyebrow scars prior to follicular transplantation to create favorable conditions for the survival and growth of transplanted follicular units. We selected fractional ablative carbon dioxide laser among various types of lasers because, in comparison to others, it exhibits significant advantages in the removal of scar tissue, promotion of collagen regeneration and restructuring, elimination of pigment deposition, improvement of blood circulation, and facilitation of the transformation of scar tissue towards normalcy, particularly for scars resulting from severe trauma healing.^12^ Regarding the results of the POSAS assessment in our study, a significant difference was observed between the patient self‐assessment and observer assessment scores in the experimental group post‐laser treatment. We attribute this discrepancy to the fact that the patients were non‐professionals who may not have fully appreciated the improvement in scars post‐laser treatment. Most of them still perceived their scars as severe, with the relief of various symptoms not being sufficiently evident. In contrast, in the observer assessment, medical professionals could objectively observe improvements in various aspects of the scars.

With the continual enhancement of eyebrow transplantation techniques and the ongoing improvement of post‐operative care protocols, traditional complications such as bleeding, infection, and wound dehiscence following eyebrow transplantation have become increasingly rare.^13^ Currently, attention should be paid to preventing post‐operative aesthetic complications, such as severe eyebrow asymmetry or eyebrow curling.^14^ Therefore, for patients with eyebrow loss accompanied by scarring, we continuously improve treatment methods based on traditional eyebrow transplantation, aiming to ensure a high follicular transplant survival rate and favorable aesthetic outcomes.

In our previous clinical experience, some patients with typical follicular transplantation showed acceptable follicle survival rates post‐operatively. While they frequently expressed concerns about curly hair and noticeable bilateral asymmetry in the eyebrows. We speculate that this may be related to the thick and firm texture of the traumatic scars. In normal cases, the establishment of the direction of eyebrow hair is closely related to the angles and directions of the holes created during surgery. However, when patients have eyebrow scars, the hard and thick texture of the scars can lead to internal tunnel distortion during hole creation, resulting in the bending of transplanted hair or bilateral eyebrow asymmetry.

There were also several limitations in our study. Firstly, the number of patients included was limited. A larger sample size would enhance the persuasiveness of the results. Secondly, as this study was a controlled trial, there were slight differences in the selection of donor areas between the two groups, and there may have been variations in post‐operative care among patients. These factors could potentially introduce some degree of error in the final results.

## 5 CONCLUSION

This study was a prospective comparative study. For patients with post‐traumatic eyebrow scars accompanied by loss, this study achieved a higher follicle survival rate and good eyebrow appearance post‐eyebrow transplantation using Follicular Unit Extraction combined with fractional ablative carbon dioxide laser treatment.

## AUTHOR CONTRIBUTIONS

P.X., Q.Y., L.G., and Y.Y. designed and performed the research. X.F. and B.S. conceived of and supervised the research. W.D., T.L., and X.Z. analyzed the data. P.X., Q.Y., and L.G. wrote the paper. All authors have read and approved the final manuscript.

## FUNDING INFORMATION

No funding was received for this study.

## CONFLICT OF INTEREST STATEMENT

The authors have nothing to disclose.

## Data Availability

The data that support the findings of this study are available on request from the corresponding author. The data are not publicly available due to privacy or ethical restrictions.
